# Sympathetic Ophthalmia Two Weeks After 23-Gauge Vitrectomy

**DOI:** 10.1186/s12348-020-00206-2

**Published:** 2020-06-26

**Authors:** Bahaeddin B.E. EL Khatib, Menka S.M.P. Patel, Alexander A.H. Hacopian, Monica M.D. Dalal, H. Nida H.N.S. Sen, Marena M.P. Patronas

**Affiliations:** 1 Department of Ophthalmology, George Washington University, 2150 Pennsylvania Ave NW, Washington, DC 20037 USA; 2 National Eye Institute, National Institute of Health, Bethesda, MD USA

## Case report

Sympathetic ophthalmia (SO) is a rare disease that presents as a bilateral, diffuse, granulomatous panuveitis. Sympathetic ophthalmia is a clinical diagnosis with a history of penetrating ocular injury in the inciting eye and the presence of panuveitis in the sympathizing eye. Though early enucleation is believed to minimize the risk, there have been reports of SO even after the enucleation of inciting eyes. The possible association between vitrectomy and SO has been initially proposed by Gass [9] and later studied extensively in a large cohort in the UK with an estimated SO risk of 1 in 799 vitrectomies [11]. There have been several case series and reports of SO following vitrectomy; however, only three documented cases of SO following vitrectomy without the use of silicone oil with an onset of SO ranged between 4 weeks and 2 months. We present a patient with SO in the sympathizing eye presenting 16 days after an uncomplicated 23-gauge (23G) sutureless pars plana vitrectomy (PPV) without the use of silicone oil.

**Fig. 1:**
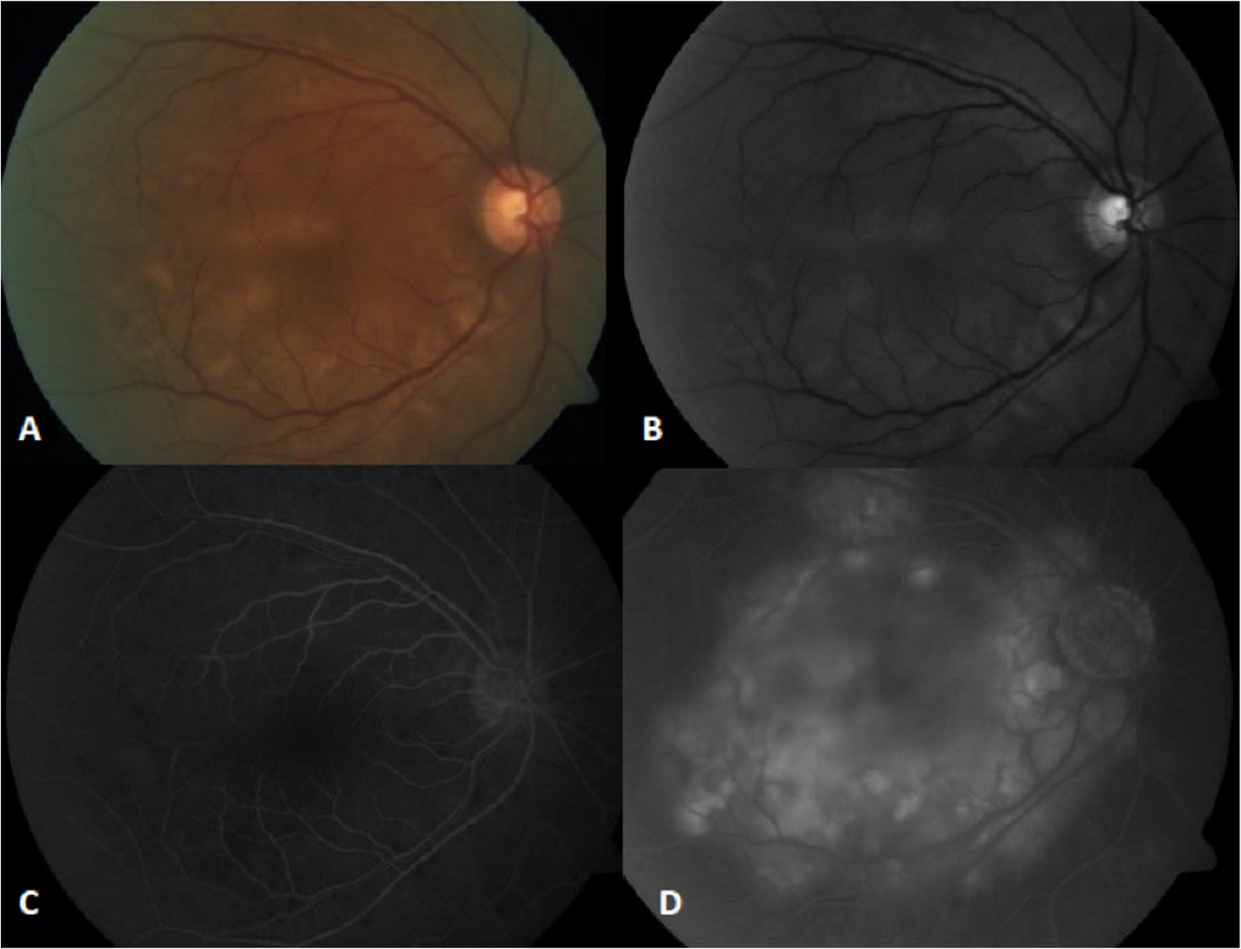
**a** Color fundus photograph of the right eye. **b** The red free of the right eye shows hypopigmented/yellowish areas. **c** Fluorescein angiogram in the early phase demonstrates patchy hypofluorescence early and **d** late leakage and pool corresponding to areas of yellow hypopigmentation

**Fig. 2:**
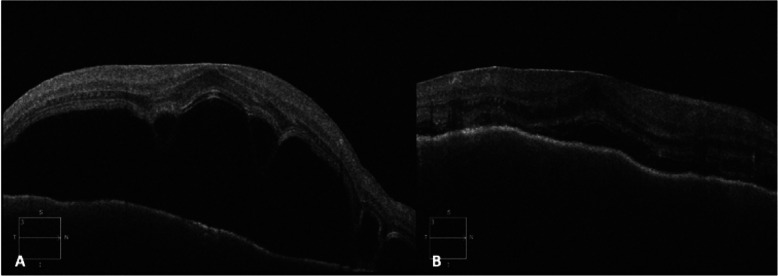
**a** Optical coherence tomography of the right eye demonstrating a large macular serous detachment. **b** Optical coherence tomography of the right eye demonstrating improvement in macular serous detachment after acetezolamide

A 60-year-old Indian male presented with macula-off retinal detachment with multiple tears in his left eye (OS) and underwent 23G pars plana vitrectomy, endodiathermy, endodrainage, endolaser photocoagulation, and 15% octafluoropropane gas (C3F8) OS. His past medical history was significant for thyroid cancer and total thyroidectomy, and past ocular history was significant for cataract extraction and posterior chamber intraocular lens (PCIOL) implantation OS 9 years ago with no history of intraocular trauma or surgery in the right eye (OD). His postoperative course was relatively unremarkable until he reported relative scotoma in the fellow eye (OD) on postoperative day 16. On examination, his visual acuity was hand-motion OD (from a baseline of 20/30-2) and counting-fingers (CF) OS from the postoperative gas bubble. Slit-lamp exam (SLE) revealed no anterior chamber (AC) cells or flare OD; rare AC cell without flare OS and dilated fundus examination (DFE) showed serous retinal detachment of the macula with no disk edema, vascular sheathing, or vitritis OD (Fig. 1), and an attached retina with approximately 70% C3F8 gas fill OS. Optical coherence tomography (OCT) confirmed a serous macular detachment with nasal pigment epithelial detachment (PED) (Fig. 2a).

The patient was initially treated with acetazolamide (Diamox) for presumptive Central Serous Chorioretinopathy (CSR) with initial improvement in subretinal fluid (SRF) and visual acuity (OD: 20/200) (Fig. 2b). However, 2 weeks later, the patient developed new bilateral granulomatous panuveitis with bilateral 1+ conjunctival injection, AC cell and flare, and 2+ vitreous cells with haze OD greater than OS and disk edema OS. Quantiferon gold test was negative. No systemic associations were found, including sensorineural hearing loss, tinnitus, fever, headache, vertigo, vitiligo, poliosis, or alopecia. In addition, there was no ocular depigmentation appreciated on the exam. These findings along with a history of surgery preceding the uveitis suggest sympathetic ophthalmia and not Vogt-Koyanagi-Harada (VKH). He was diagnosed with sympathetic ophthalmia, and he was started on high-dose prednisone (80 mg PO daily). Peripheral retinal ischemia and a rhegmatogenous retinal detachment (RRD) developed in the right eye, and so, he was converted to intravenous pulse steroids for 3 days in order to quiet the inflamed eye prior to retinal detachment repair.

**Fig. 3:**
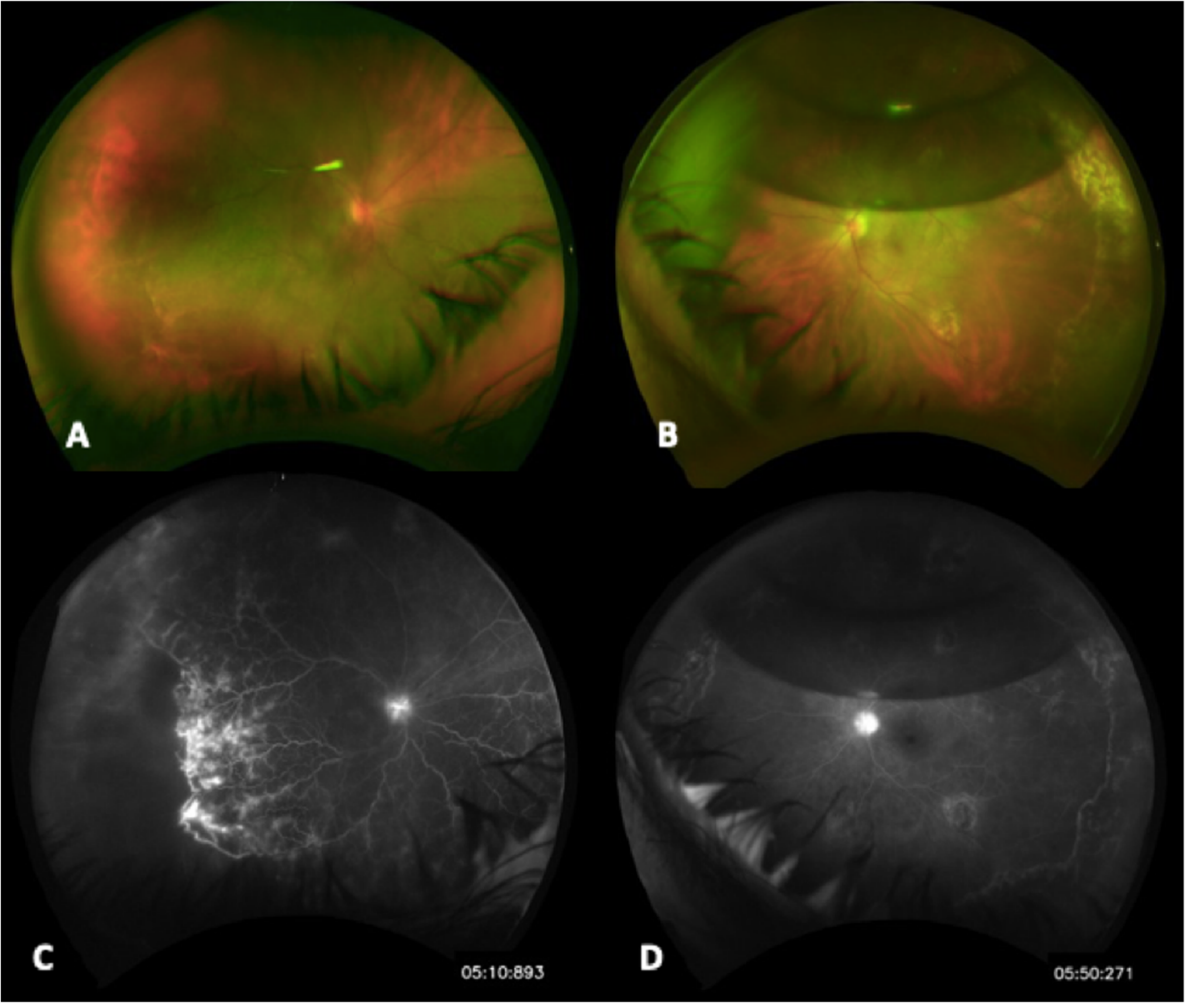
**a** Color fundus photograph of the right eye with inferotemporal RRD macula off. **b** Color fundus photograph of the left eye with macula attached and periphery with laser scars more evident temporally. Mid-periphery chorioretinal scar from drainage and superior C3F8 bubble with 40% fill. **c** Fluorescein angiography of the right eye late images demonstrating disk hyperfluorescence, temporal nonperfusion, and vascular staining and leakage in the area of the retinal detachment. Mild macular hyperfluorescence is present. **d** FA of the left eye, the late images demonstrating disk staining, and no other abnormal hyperfluorescence other than staining of postsurgical laser scars

A fluorescein angiography (FA) performed prior to starting intravenous (IV) steroids showed notable hyperfluorescence of the optic nerves in both eyes and significant inferotemporal nonperfusion with pruning of vessels temporally OD (Fig. 3). A combined cataract extraction, PCIOL insertion, and retinal detachment repair with PPV, endolaser, air-fluid exchange, and C3F8 gas were performed on the right eye approximately 6 weeks after the left eye retinal detachment repair was performed.

**Fig. 4:**
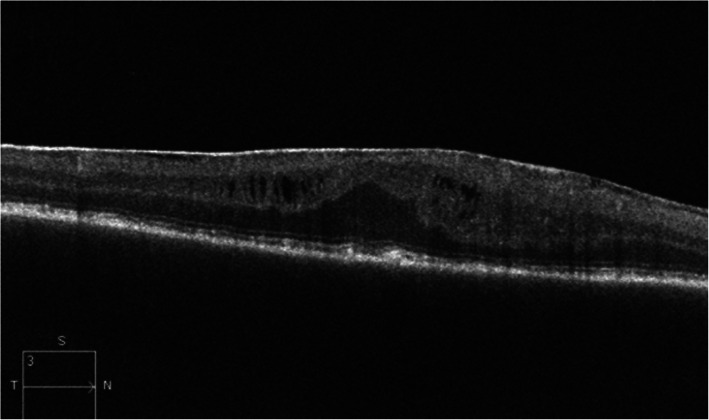
OCT right eye, 1 year after the initiation of treatment for panuveitis from SO. ERM without significant inner retinal wrinkling, loss of foveal contour, mild intraretinal edema, and significant RPE irregularity

Over the next year, the patient was managed at the National Institute of Health (NIH) with systemic immune suppression, topical, and local anti-inflammatory treatment to the left eye. At the 1-year follow-up, the patient’s vision is OD: 20/200 and OS: 20/25-1. An OCT OD shows an epiretinal membrane (ERM) with loss of foveal contour, mild intraretinal edema, and significant irregularity of the retinal pigment epithelium (RPE) (Fig. 4).

Sympathetic ophthalmia has been associated with specific major histocompatibility complex (MHC), and patients are likely to express human leukocyte antigen (HLA) DR4 (closely related to HLA-DQw3 and HLA-DRw53) phenotype [2]. These specific marker associations suggest a role for immune dysregulation, increased susceptibility, and increased severity associated with pathogenesis [2].

Although the etiology is not clearly understood, it encompasses autoimmunity and cell-mediated immune mechanisms. It has been postulated to be a delayed-hypersensitivity reaction [2–4]. Interestingly, our patient was positive for HLA-DR4.

The eye is considered an immune-privileged site, and SO requires sequestered ocular antigens to be exposed systemically, likely from a scleral perforation, whether surgical or traumatic [5–8, 10, 12]. Though a link between SO and vitrectomy was made in the 1980s, only a few cases of SO have been reported following uncomplicated vitrectomy without the use of silicone oil or without antecedent trauma or endophthalmitis. In most cases, the average time of onset of SO was 2 months to 1 year [13]. There are only two cases of SO [1, 13] that occurred 5 weeks to 2 months after small incision PPV without the use of silicone oil for tamponade. To the best of our knowledge, our case presented here represents the earliest case of SO following 23G sutureless vitrectomy for RD without silicone oil and antecedent trauma with an onset at 16 days following surgery. Sympathetic ophthalmia should be considered in cases of new onset posterior uveitis soon after vitrectomy.

## Data Availability

Data sharing is not applicable to this article as no datasets were generated or analyzed during the current study.

## Abbreviations

23G: 23 Gauge
AC: Anterior chamber
C3F8: Octafluoropropane gas
CSR: Central serous chorioretinopathy
DFE: Dilated fundus examination
FA: Fluorescein angiography
HLA: Human leukocyte antigen
IV: Intravenous
MHC: Major histocompatibility complex
NIH: National Institute of Health
OCT: Optical coherence tomography
OD: Right eye
OS: Left eye
PCIOL: Posterior chamber intraocular lens
PED: Pigment epithelial detachment
PPV: Pars plana vitrectomy
RPE: Retinal pigment epithelium
RRD: Rhegmatogenous retinal detachment
SLE: Slit-lamp exam
SO: Sympathetic ophthalmia
SRF: Subretinal fluid
VKH: Vogt-Koyanagi-Harada
